# The Triple-Negative Breast Cancer Database: an omics platform for reference, integration and analysis of triple-negative breast cancer data

**DOI:** 10.1186/s13058-014-0490-y

**Published:** 2014-12-04

**Authors:** Rajesh Raju, Aswathy Mary Paul, Vivekanand Asokachandran, Bijesh George, Lekshmi Radhamony, Meena Vinaykumar, Reshmi Girijadevi, Madhavan Radhakrishna Pillai

**Affiliations:** Computational Biology Group, Cancer Research Program-9, Rajiv Gandhi Centre for Biotechnology, Poojappura, Thiruvananthapuram, 695014 Kerala India

## Abstract

No abstract

**Electronic supplementary material:**

The online version of this article (doi:10.1186/s13058-014-0490-y) contains supplementary material, which is available to authorized users.

Clustering the transcriptomic profile of 587 triple-negative breast cancer (TNBC) cases extracted from 21 breast cancer microarray datasets relying on the lack of transcript-level expression of estrogen receptor (ER), progesterone receptor (PR) and human epidermal growth factor receptor 2 (Her2), Lehmann and colleagues have categorized the TNBCs into seven subgroups [1]. From more than 1,000 clinical samples that were characterized as TNBCs by immunohistochemistry, we report the first compendium of molecular expression-level alterations as a value-added resource for extended research. This open-access manually curated resource, the Triple Negative Breast Cancer Database (TNBCDb) [2], currently hosts 144 microRNA, 2,696 mRNA, 106 protein and 13 post-translational modification alterations in TNBC tissues.

The TNBC tissues are categorized into lymph node-positive, lymph node-negative and lymph node metastatic tissues, or otherwise as TNBC-not specified. TNBCDb hosts experimentally reported alterations in these tissues compared with ER^+^, ER^+^PR^+^, Her2^+^, ER^+^PR^+^Her2^−^, ER^−^PR^−^Her2^+^, luminal A, luminal B and non-TNBC (if not specified) or matched adjacent normal, unmatched normal breast or parenchyma tissues as analyzed. The ethnicity/origin as provided by the authors/deduced, the frequency of observation of the molecular alterations in terms of the number of patient samples analyzed per study, fold values of expression, experiment platform used and the reference to corresponding research articles are provided for the curated records.

TNBCDb also hosts the comparative molecular profile of 39 TNBC cell lines compared among themselves as well as with 55 non-TNBC cell lines as a tool for selection of appropriate cell lines for specific studies on the basis of their molecular background. Effective visualization of genes differentially regulated in TNBC tissues and cell lines and, further, the signaling pathways [3],[4], biological processes, molecular functions, cellular localization [5], protein–protein interactions [6], microRNA targets and RNA-level co-expressed genes [7] that are associated with each type of molecule, is enhanced through unique features designated the ‘TNBCDb viewer’ and the ‘Network viewer’ (Figure 1).

**Figure 1:**
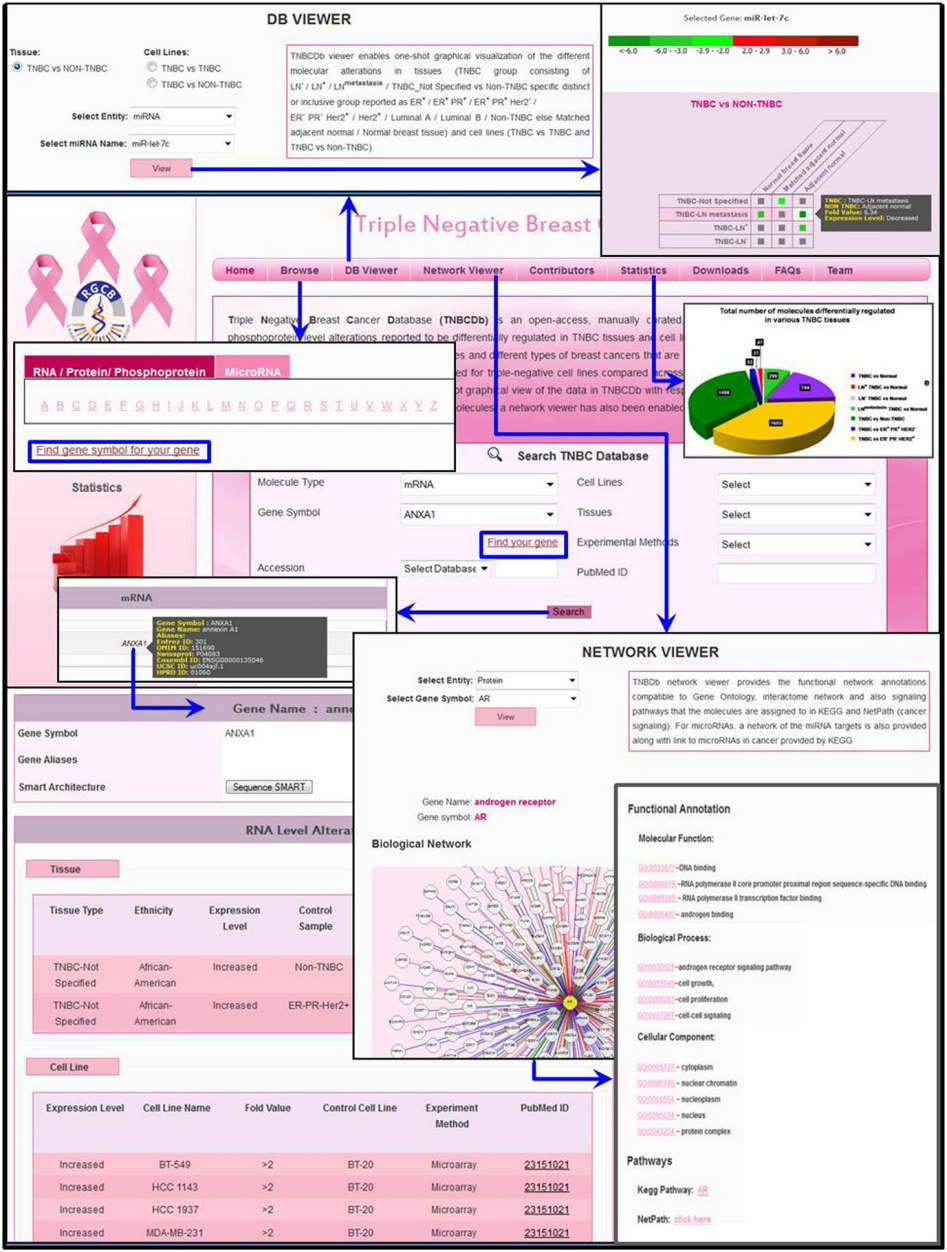
**Navigation through the Triple-Negative Breast Cancer Database.** The Triple Negative Breast Cancer Database (TNBCDb) has features enabled to search molecule types by molecule gene symbol and different accession numbers (Entrez Gene, OMIM, SwissProt and MiRbase IDs). To help researchers search genes by gene symbol, a ‘find your gene’ feature has been incorporated that allows selection of molecules from the browse page or helps find the corresponding gene symbols for your molecule. For each of the microRNAs, mRNAs, proteins or phosphoproteins, their differential regulation in tissues as well as the cell lines is provided as available. The TNBCDb viewer has been enabled to facilitate visualization of the regulation of molecules in triple-negative breast cancer (TNBC) tissue(s) versus different types of non-TNBC tissues and for molecules in one TNBC cell line with another TNBC cell line and with different non-TNBC cell lines highlighting their fold-change values. The network viewer enables the integrated view of the interactions, co-expressions, biological processes, molecular function, cellular compartments and the biological pathways that are reported to be associated with each of the molecules in TNBCDb. Further, researchers can also search by tissue type, cell line name, experiment method and PubMed IDs as individual or multiple queries. The TNBCDb data in the complete form are available for free download [8]. ER, estrogen receptor; Her2, human epidermal growth factor receptor 2; LN, lymph node; PR, progesterone receptor.

Considering the heterogeneity of breast cancers, we believe the TNBCDb will serve as a platform for selection of therapeutically relevant molecular entities from the tissue and cell line information and also for the selection of appropriate cell lines for evaluation of therapeutic targets in the direction of personalized therapy. We request suggestions from the scientific community to improve and keep this resource up to date with more information/clinical parameters through an online portal [9]. We believe that this initiative will help us to maintain TNBCDb as a global reference, integration and analysis platform for TNBC.

## Authors’ original submitted files for images

Below are the links to the authors’ original submitted files for images. Authors’ original file for figure 1

## Abbreviations

ER: Estrogen receptor
Her2: Human epidermal growth factor receptor 2
PR: Progesterone receptor
TNBC: Triple-negative breast cancer
TNBCDb: Triple Negative Breast Cancer Database
