# Supplementary figures and images for: The Triple-Negative Breast Cancer Database: an omics platform for reference, integration and analysis of triple-negative breast cancer data
Source: Breast Cancer Res. 2014 Dec 4;16:490. doi: 10.1186/s13058-014-0490-y (PMC4303197; doi:10.1186/s13058-014-0490-y)

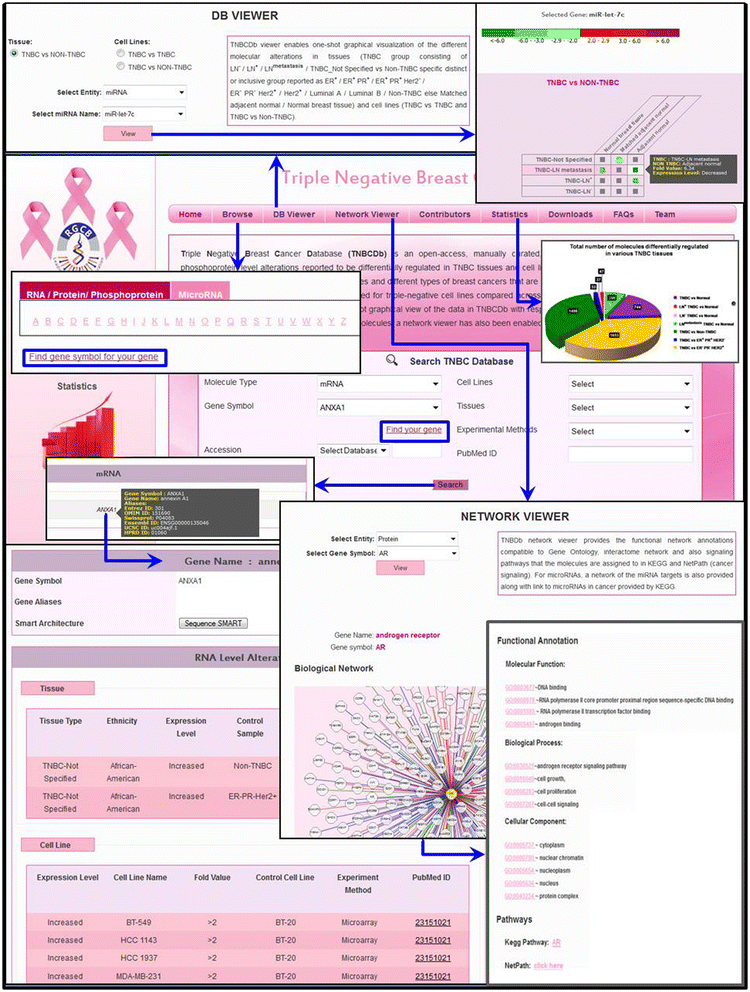

Supplement: Supplementary file 1 — Authors’ original file for figure 1 [file 13058_2014_490_MOESM1_ESM.gif]
